# Genetic diversity of enteric viruses responsible of gastroenteritis in urban and rural Burkina Faso

**DOI:** 10.1371/journal.pntd.0012228

**Published:** 2024-07-08

**Authors:** Ange Oho Roseline Badjo, Sandra Niendorf, Sonja Jacobsen, Arsène Zongo, Andreas Mas Marques, Ann Christin Vietor, Nongodo Firmin Kabore, Armel Poda, Satouro Arsène Some, Aminata Ouattara, Soumeya Ouangraoua, Grit Schubert, Tim Eckmanns, Fabian H. Leendertz, Essia Belarbi, Abdoul-Salam Ouedraogo

**Affiliations:** 1 Laboratory of Emerging and Re-emerging Pathogens, Nazi Boni University, Bobo Dioulasso, Burkina Faso; 2 Robert Koch Institute, Berlin, Germany; 3 Centre MURAZ, Bobo-Dioulasso, Burkina Faso; 4 Department of Infectious Diseases, University Hospital Souro Sanou, Bobo-Dioulasso, Burkina Faso; 5 Bacteriology and Virology Department Souro Sanou University Hospital, Bobo-Dioulasso, Burkina Faso; 6 Helmholtz Institute for One Health, Greifswald, Germany; University of Sussex, UNITED KINGDOM

## Abstract

**Background:**

Viral gastrointestinal infections remain a major public health concern in developing countries. In Burkina Faso, there are very limited updated data on the circulating viruses and their genetic diversity.

**Objectives:**

This study investigates the detection rates and characteristics of rotavirus A (RVA), norovirus (NoV), sapovirus (SaV) and human astrovirus (HAstV) in patients of all ages with acute gastrointestinal infection in urban and rural areas.

**Study design & Methods:**

From 2018 to 2021, stool samples from 1,295 patients with acute gastroenteritis were collected and screened for RVA, NoV, SaV and HAstV. Genotyping and phylogenetic analyses were performed on a subset of samples.

**Results:**

At least one virus was detected in 34.1% of samples. NoV and SaV were predominant with detection rates of respectively 10.5 and 8.8%. We identified rare genotypes of NoV GII, RVA and HAstV, recombinant HAstV strains and a potential zoonotic RVA transmission event.

**Conclusions:**

We give an up-to-date epidemiological picture of enteric viruses in Burkina Faso, showing a decrease in prevalence but a high diversity of circulating strains. However, viral gastroenteritis remains a public health burden, particularly in pediatric settings. Our data advocate for the implementation of routine viral surveillance and updated management algorithms for diarrheal disease.

## Author summary

Diseases of the gastrointestinal tract can be caused by a variety of pathogens, such as bacteria, viruses, fungi or parasites. Viruses play a particularly important role, especially in low-income countries, where viral gastroenteritis leads to high morbidity and mortality, particularly among children and the elderly. As part of the African Network for Improved Diagnostics, Epidemiology and Management of Common Infectious Agents (ANDEMIA), patients of all ages from four African countries with symptoms of acute gastroenteritis were tested for the most common viral, bacterial and parasitic pathogens. Samples from Burkina Faso that tested positive for noroviruses, rotaviruses, sapoviruses or astroviruses were further genotyped and characterized in the present study. In this study we described for the first time the detection of rare rotavirus and astrovirus strains circulating in Burkinabe patients. The knowledge about the circulating virus variants is essential to develop suitable vaccines and adequate pharmaceuticals.

## Introduction

Gastroenteritis represents a major public health issue and cause of morbidity and mortality worldwide [1]. A large variety of enteric pathogens agents, such as bacteria, parasites, fungi and viruses can cause gastrointestinal infections [2]. However, viral etiologies involving rotavirus A (RVA), norovirus (NoV), human astrovirus (HAstV), adenovirus (AdV), and sapovirus (SaV) are more common [3].

Burkina Faso (BFA) is a low-income, landlocked country in West Africa with a tropical to semi-arid climate with both rainy and dry seasons [4]. Until 2013, RVA was the most prevalent enteric virus observed in symptomatic patients followed by other viral agents, including AdV, genogroup II (GII) NoV, SaV, HAstV and genogroup I (GI) NoV [5]. However, the introduction of the RVA vaccine following WHO recommendations changed the epidemiology of viral gastroenteritis, NoV becoming the leading viral etiology [6].

*Rotaviruses* are non-enveloped viruses with a segmented double stranded ribonucleic acid (RNA) genome. They belong to the *Reoviridae* family and are classified into 9 groups based on the viral protein (VP) 6 [7]. RVAs infect a wide range of vertebrates and are one of the most important pathogens responsible of infantile gastroenteritis, with 128,500 deaths in children under 5 years old, especially in low- and middle-income countries (LMIC) [8]. RVA are classified based on the variability of the VP7 (G type) and the VP4 (P type) glycoproteins. Currently, 42 G types and, 58 P types have been described [7]. Prior to the vaccine introduction, RVA was the leading cause of hospitalizations related to severe childhood diarrhea, with detection rates up to 63% [5]. The RotaTeq vaccine was introduced in October 2013 in BFA’s routine immunization program with a coverage >90% since 2014. It is administered to infants at 2, 3, and 4 months of age and covers five human RVA genotypes: G1, G2, G3, G4, and P[8] [9].

*Noroviruses* belong to the *Calciviridae* family. They are small non-enveloped viruses with a positive single-stranded (+ss) RNA genome divided in three Open Reading Frames (ORF). They are the leading cause of gastroenteritis in children and are associated with an estimated 200,000+ deaths per year [10]. NoV are classified into 10 genogroups (GI to GX) and more than 40 genotypes based on the capsid gene [11]. They infect a large variety of mammals; however, most of human norovirus strains belong to genogroups GI and GII [11].

*Sapoviruses* belong to the *Caliciviridae* family. They are non-enveloped viruses with a +ssRNA genome [12]. Currently, 4 human SaV genogroups are classified into 17 genotypes based on the capsid gene [12]. Coinfections with other enteric viruses have been noted in acute gastroenteritis outbreaks in humans [13]. A systematic review of studies in LMIC showed an average detection rate of 6.5% and highlighted the need for a better understanding of its role in diarrheal diseases [13].

Astroviruses belong to the *Astroviridae* family. They are small non-enveloped viruses with a +ssRNA genome [14]. They have been isolated from stool from a large variety of species (humans and domestic, wild and marine mammals), and were mostly associated with gastroenteritis [14]. Their classification is complex as it was initiated on the basis of the host range and was updated by recent phylogenetic analyses. Classical HAstV comprise genotypes 1 to 8. Two novel groups of HAstV divergent from the classical ones have been identified, namely MLB (Melbourne) and VA/HMO (Virginia/Human-Mink-Ovine-like) [14].

Correct diagnosis, epidemiological and genetic surveillance play a decisive role in containing the spread of infectious diseases and reducing public health risks. However, the limited technical resources of many laboratories in BFA do not allow routine screening of most viruses, contributing to the knowledge gap.

Within the framework of the African Network for Improved Diagnostics, Epidemiology and Management of Common Infectious Agents (ANDEMIA) [15], we aimed to investigate the role of enteric viruses in acute gastroenteritis (AGE) patients of all ages from urban and rural areas in BFA and to further characterize the genetic diversity of the circulating strains.

## Methods

### Ethics statement

The study adheres to the tenets of the Declaration of Helsinki, as well as national legislation and ethical standards. Approval by the national ethics committees has been obtained for the ANDEMIA study: Comité d’Ethique pour la Recherche en Santé (approval number 2017–5–057) in Burkina Faso and Ethikkommission—Ethikausschuss am Campus Virchow-Klinikum, Charité (approval number EA2/230/17) in Germany.

### Study population

This study was conducted from February 2018 to December 2021 within the ANDEMIA network [15]. A total of 1,295 patients of all ages with acute gastrointestinal infection were enrolled in urban and rural sentinel sites in Burkina Faso (S1 Fig). Cases of acute gastrointestinal infection were defined as patients with diarrhea (3 loose or liquid stool in the last 24 hours). Chronic cases (onset of symptoms > 4 weeks) and patients admitted to hospital for more than 48 hours were excluded. Enrolments took place at the university hospital of Souro Sanou in Bobo Dioulasso in the “Hauts-Bassins” region and in health centers around Dano and Dissin in the “Sud-Ouest” region. Participants provided written informed consent and answered a clinical and socio-economical questionnaire. Stool samples or rectal swabs were collected at enrolment.

### Screening for gastrointestinal viruses

Nucleic acid extracts were obtained from stool samples or rectal swabs using IndiSpin Pathogen Kit (Indical, Germany) following the manufacturer´s instructions. Detection of viral nucleic acid was performed using the FTD viral gastroenteritis kit (Siemens Health Care, Germany) following the manufacturer´s instructions. This multiplex RT-PCR allows the detection of NoV GI and GII, HAstV, RVA (including the attenuated vaccine strains Rotateq and Rotarix), SaV and AdV. According to manufacturer the diagnostic sensitivity is 100% for all targets except NoV GII for which it is 97.9%. Diagnostic specificity is 100% for all targets. The overall accuracy value for FTD Viral gastroenteritis has been evaluated to 99.9%. The screening results for AdV won’t be shown here. Positive extracts were stored at -80°C until further processing.

### Genotyping and phylogenetic analyses

Determination of RVA G and P-types was done as previously described [16]. NoV and SaV, positive samples were genotyped based on the RNA dependent RNA polymerase (RdRp) gene (ORF1) and the capsid gene (ORF2, P2 region) as previously described [17]. HAstV positive samples were re-tested with a PCR amplifying a fragment of the RdRp gene (ORF1) (>600bp) and a pan-specific HAstV semi-nested RT-PCR [18]. Samples found negative with the initial ORF1 PCR were further tested with a PCR covering a short fragment of the RdRp gene (~180 bp) [18]. Samples assigned to HAstV1-8 or MLB1-3 genotypes in ORF1b were further characterized. For the classical genotypes, fragments starting in ORF1b and ending in ORF2 were generated (with ~334 nucleotides corresponding to ORF2). For MLB genotypes, ~800 bp fragments from ORF2 were amplified. All amplicons were purified using EXOSAP-IT (Affymetrix Inc. USB Products, Cleveland, USA) and used for direct Sanger-sequencing (GenBank accession numbers available in S2 Table). Sequences alignments were done with MAFFT algorithm in Geneious Prime 2021 (Biomatters Ltd, New Zealand). The best fit substitution model was calculated and modelling of Maximum-Likelihood trees or Neighboor-Joining trees was done with bootstrap test of 1000 replicates using the Molecular Evolutionary Genetics Analysis (MEGA) software version 11.0.11. The obtained phylogenetic trees were edited using the Interactive Tree Of Life version 6.8.2 online tool and Inkscape software. Phylogenetic analyses of the HAstV ORF2 region were done to identify putative intergenotype recombinant viruses.

### Statistical analyses

Statistical analyses were performed using Stata/MP 15.1 (StataCorp, Texas, USA) and R (v4.3.0). Proportions were compared using the Pearson’s Chi-square and Fisher’s exact tests. P-values < 0.05 were considered to be statistically significant.

## Results

### Detection rate of enteric viruses and risk factors

More than half of the patients in our study were male (55.3%) and children under 5 years were the predominant age group (88.7% of the study population). The participants mean age was 4.5 years (0 to 85 years). The majority of participants (75.7%) resided in a rural area (Table 1). Among the socio-demographic and clinical features tested, age, fever (measured at enrollment or self-reported in the 10 days prior enrollment) and hospitalization status were found to be significantly different between enteric viruses’ negative and positive groups.

**Table 1:** Demographic and clinical characteristics of study participants *versus* positivity rate for enteric viruses.

Symptomatic patients	Enteric viruses’ status			
	Positive N (%)	Negative N (%)		p-value
**Gender (n = 1293)**				
Male	247(56%)	469 (55%)		0.7
Female	194 (44%)	383 (45%)		
**Age groups (n = 1295)**				
< 5 years	423 (96%)	725 (85%)		<0,001*
5–15 years	5 (1%)	42 (5%)		
>15 years	13 (3%)	87 (10%)		
**Residency (n = 1295)**				
Urban	102 (23%)	213 (25%)		0.5
Rural	339 (77%)	641 (75%)		
**Fever (n = 1295)**				
Yes	315 (71%)	656 (77%)		0.03*
No	126 (29%)	198 (23%)		
**Nausea or vomiting (n = 1290)**				
Yes	182 (41%)	370 (40%)		0.5
No	258 (59%)	480 (56%)		
**Presence of blood in stool (n = 1295)**				
Yes	405 (92%)		788 (92%)	0.8
No	36 (8%)		66 (8%)	
**Weight loss (n = 1281)**				
No	214 (49%)		449 (53%)	0.2
Yes	221 (51%)		397 (47%)	
**Comorbidities (n = 1282)**				
No	432 (99%)		835 (99%)	1
Yes	5 (1%)		10 (1%)	
**Rapid malaria test (n = 1180)**				
Negative	357 (87%)		656 (85%)	0.4
Positive	53 (13%)		114 (15%)	
**Hospitalization (n = 1291)**				
No	236 (54%)		380 (45%)	0,002*
Yes	204 (46%)		471 (55%)	

* Indicates a statistically significant p-value (Pearson χ2 test)

**Fig 1:**
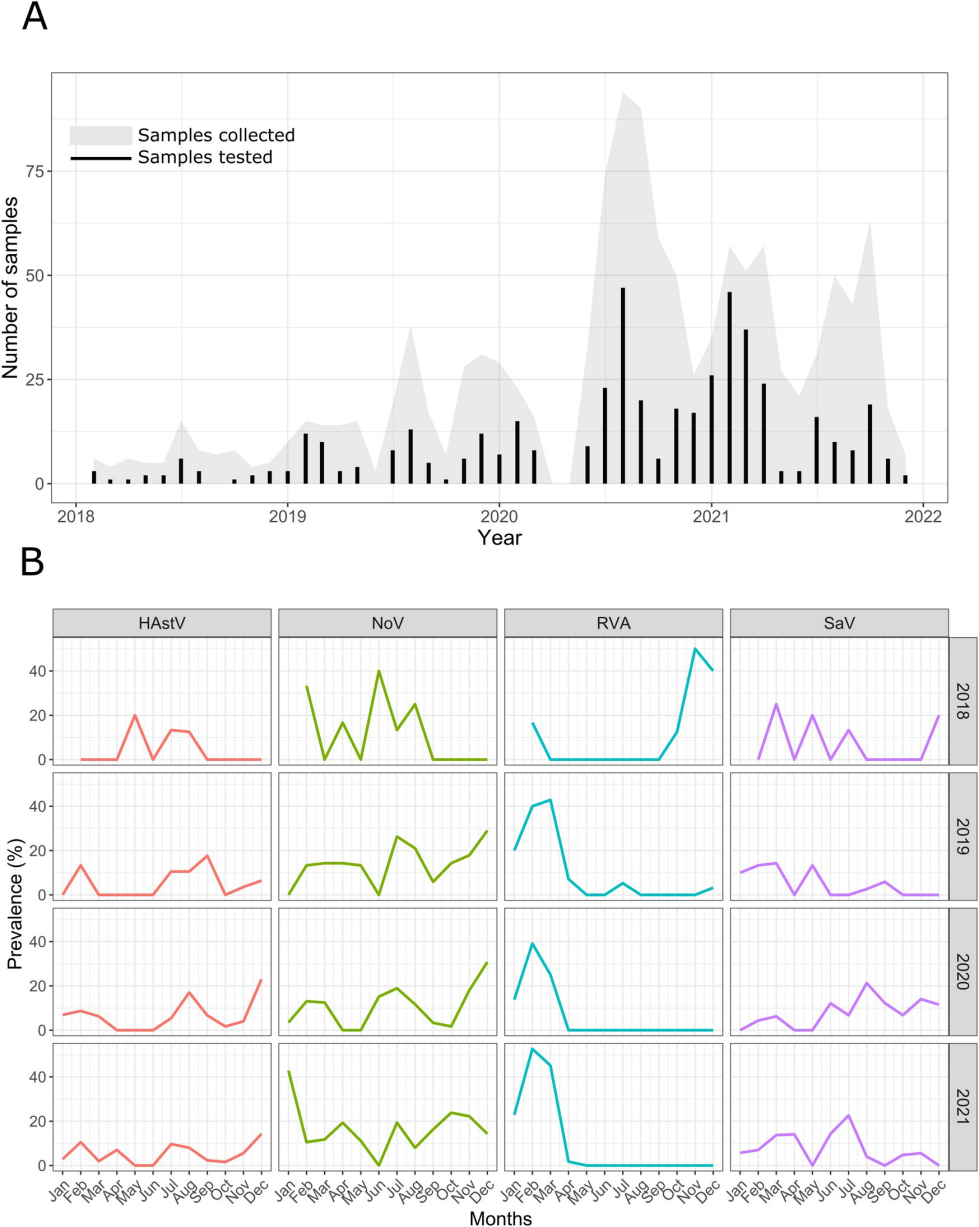
ANDEMIA patients sampling and screening results. A. Number of samples tested per enrolment month and year are indicated with a grey area (min = 0, max = 94, median = 18), the number of samples positive to one or more of the tested enteric viruses (NoV, SaV, RVA, HAstV) are indicated with black bars (min = 0, max = 47, median = 6). B. Prevalence of HAstV, NoV, RVA and SaV in 2018–2021. No samples were collected in April and May 2020 due to the COVID-19 confinement measures (Data available in S1 Table).

The number of stool samples collected per month (Fig 1A) varied from a minimum of 0 during the COVID-19 confinement measures in April and May 2020 to a maximum of 94 in August 2020 (median = 18).

A total of 441 (34.1%) samples were positive to at least one of the tested viruses. NoV GII was the most frequently detected virus (10.5%), followed by SaV (8.8%) and RVA (8.4%). HAstV and NoV GI were detected at lower rates (6.8% and 3.6% respectively) (Fig 1B). We did not observe any seasonal pattern in the detection rates of the studied viruses between 2018 and 2021 except for RVA which peaked in the colder dryer season (December to March) (Fig 1B). Among the positives, we mainly observed infections with only one virus (n = 391). Co-infections were less frequent, with 2 viruses detected in 47 samples and 3 viruses detected in 3 samples. The most common viral combinations found were RVA/SaV, HAstV/SaV and HAstV/NoV GII. HAstV (n = 26) and SaV (n = 25) were predominant in co-infected samples (S3 Table).

Children under 5 years were more susceptible to enteric viruses than all other age groups (Tables 1 and S4), and had significantly higher odds of positivity than other age groups (OR = 3.9) as shown by a multivariable risk factor analysis (Table 2). The residence area did not seem to have an effect on the overall enteric viruses’ positivity (Tables 1 and 2), it did however affect HAstV, RVA and SaV when examined individually (S5 Table). The multivariate analysis also revealed no association between gender, fever at enrolment (measured temperature ≥ 38°C), nausea/vomiting or abdominal pain and positivity for enteric viruses. Self-reported fever (in the last 10 days before enrollment) and hospitalization were associated to lower odds ratio (OR = 0.7).

**Table 2:** Risk factor analysis for enteric viruses’ positivity.

Characteristics		Univariable			Multivariable^§^		
		OR	95% CI	p-value	OR	95% CI	p-value
**Residence**							
	Urban	reference			reference		
	Rural	0.91	0.69–1.18	0.470	1.03	0.78–1.35	0.86
**Gender**							
	Male	reference			reference		
	Female	0.96	0.76–1.21	0.740	1.00	0.79–1.26	0.97
**Age (in years)**							
	≥ 15	reference			reference		
	5–15	0.80	0.24–2.27	0.68	0.82	0.25–2.35	0.72
	< 5	3.90	2.23–7.41	< 0.001*	3.94	2.23–7.51	< 0.001*
**Ongoing fever**							
	No	reference			reference		
	Yes	0.97	0.71–1.31	0.84	1.05	0.76–1.43	0.77
**Self-reported fever** ^**¥**^							
	No	reference			reference		
	Yes	0.74	0.57–0.95	0.02*	0.70	0.53–0.91	0.01*
**Abdominal pain**							
	No	reference			reference		
	Yes	0.76	0.58–0.98	0.04*	0.92	0.69–1.22	0.58
**Nausea/vomiting**							
	No	reference			reference		
	Yes	0.92	0.73–1.16	0.48	0.98	0.76–1.25	0.86
**Blood in stool**							
	No	reference			reference		
	Yes	1.06	0.68–1.61	0.78	1.18	0.76–1.81	0.46
**Weight loss**							
	No	reference			reference		
	Yes	1.17	0.93–1.47	0.19	1.14	0.90–1.44	0.27
**Comorbidities**							
	No	reference			reference		
	Yes	0.96	0.30–2.74	0.95	1.14	0.34–3.42	0.81
**Rapid Malaria test result**							
	Negative	reference			reference		
	Positive	0.85	0.60–1.21	0.38	0.83	0.57–1.18	0.31
**Hospitalization**							
	No	reference			reference		
	Yes	0.70	0.55–0.88	0.002*	0.71	0.55–0.91	0.01*

^**§**^ Adjusted for residence, gender and age group

* Significance at a 0.05 alpha level

^¥^ In the last 10 days before enrolment

For logistical reasons, 96 randomly selected positive samples were genotyped (36 NoV; 25 RVA; 18 SaV and 17 HAstV).

### RVA genotypes

We were able to type 20 strains which all belonged the group A. We identified four P and six G types, either in single or mixed infections (Table 3). The P[4] and P[6] types were predominant (30% each). The most prevalent G types were G12 (35%; n = 7) followed by G2 (25%; n = 5) and G1 (25%; n = 5) (Table 3). Among all combination, G12P[6] and G2P[4] were the most detected, 20% and 15%, respectively. Mixed infections were detected in two samples, one G1+G2P[4] and one G8+G12P[14] infection. In three RVA positive samples P-type could not be determined (P[nt]).

**Table 3:** Distribution of rotavirus G and P genotypes among ANDEMIA patients in BFA.

	G & P types					
RVA genotype	P[4]	P[6]	P[8]	P[14]	P[nt]	Total
**G1**	0	0	2	0	2	4 (20%)
**G2**	3	1	0	0	0	4 (20%)
**G3**	0	1	0	0	0	1 (5%)
**G8**	1	0	0	1	0	2 (10%)
**G12**	1	4	0	0	1	6 (30%)
**G29**	0	0	0	1	0	1 (5%)
**Gmix (G1+G2)**	1	0	0	0	0	1 (5%)
**Gmix (G8+G12)**	0	0	0	1	0	1 (5%)
**Total**	6 (30%)	6 (30%)	2 (10%)	3 (15%)	3 (15%)	20 (100%)

nt: not determined

Phylogenetic analysis of the VP4 and VP7 genes showed that the RVA strains analyzed here did not cluster with the RotaTeq vaccine strains and fell either in different lineages or genotypes.

**Fig 2:**
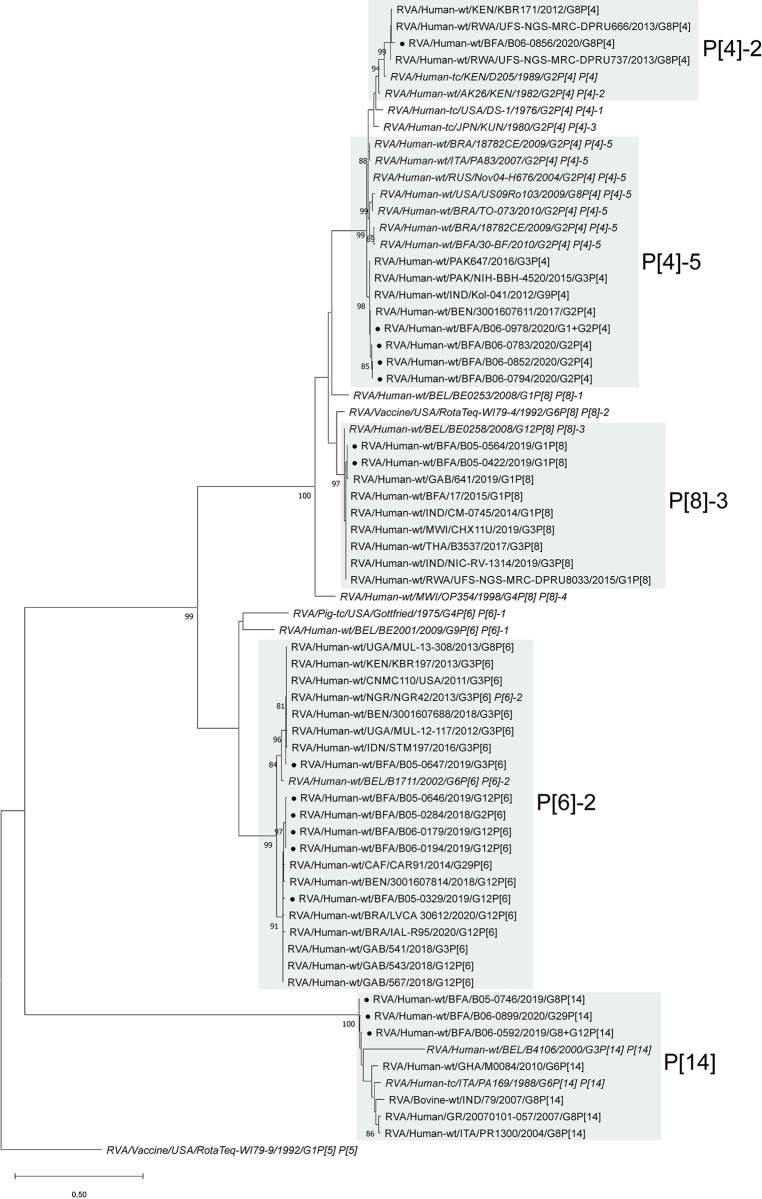
Phylogenetic analyses of RVA VP4 sequences from RVA strains from Burkina Faso. Phylogenetic analysis of VP4 gene sequences (446 nt of VP8*) of RVA strains from Burkina Faso. Closed circles (●) indicated strains from this study. Italics indicate strains added as reference for the respective genotype or lineage.

The strains B05-0422 and B05-564 are of P[8] lineage 3, clustering with strains from different African countries, India and Thailand (Fig 2). Most P[4] strains were of lineage 5, with the closest strains in GenBank being from Benin, India and Pakistan. One lineage 2 strain, B06-0856, had the highest relation to other G8P[4] strains from Africa. All P[6] strains were of lineage 2. The G12P[6] and G2P[6] strains shared almost identical sequences, indicating possible reassortment events. They clustered with other G12P[6] strains but also G3P[6] and G29P[6]. The G3P[6] strain B05-0647 shared the highest homology with other G3P[6] strains and a G8P[6] strain from Uganda (MUL-13-308). All P[14] strains from this study were distinct (<95% identity) from sequences published on GenBank. However, they shared >99% identity among each other, including the G29P[14] strain B06-0899, which implies possible reassortment (Fig 2).

**Fig 3:**
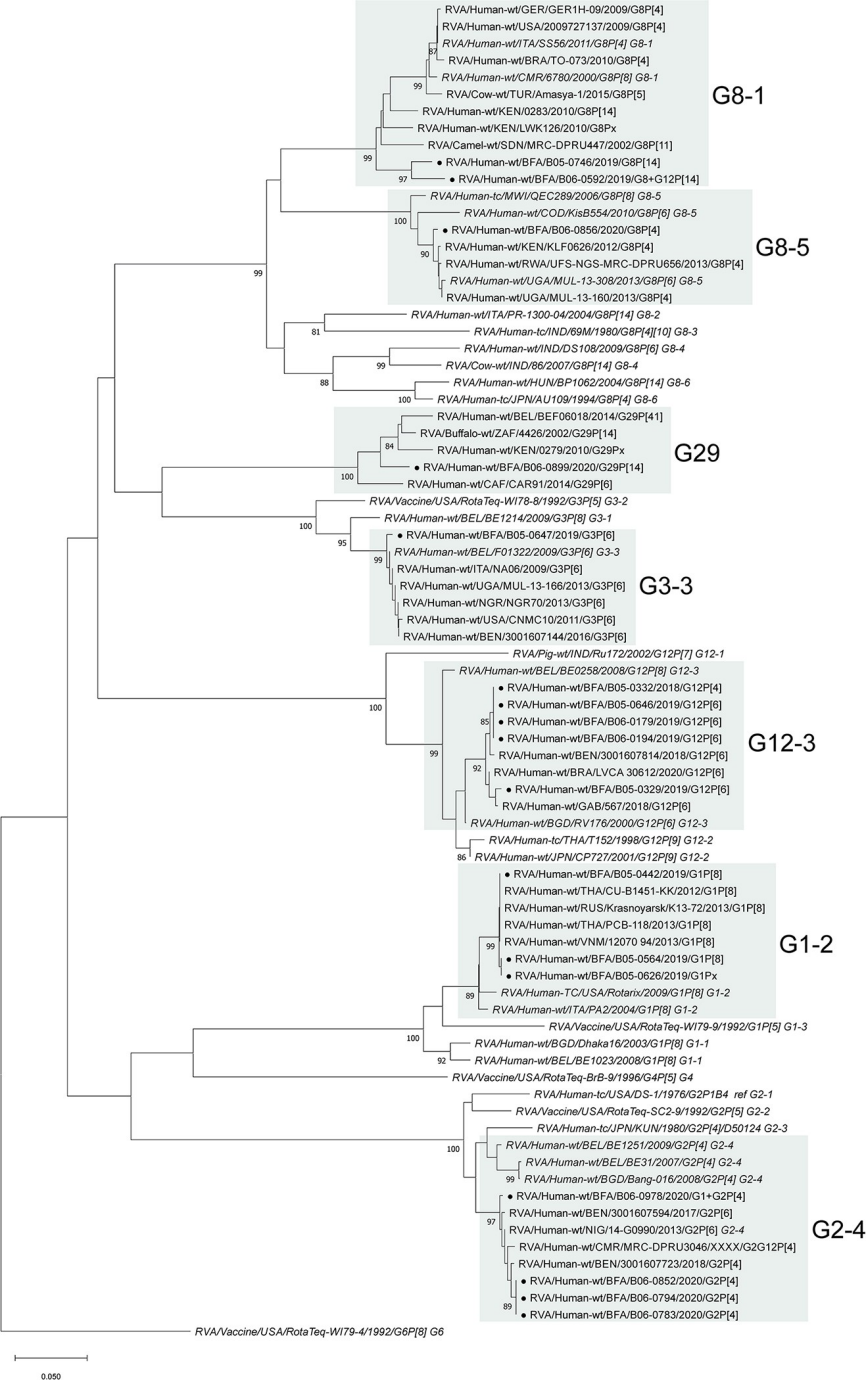
Phylogenetic analyses of RVA VP7 sequences from RVA strains from Burkina Faso. Phylogenetic analysis of VP7 gene sequences (572 nt) of RVA strains from Burkina Faso. Closed circles (●) indicated strains from this study. Italics indicate strains added as reference for the respective genotype or lineage.

Phylogenetic analysis of the VP7 gene also showed no G1 strains from this study sharing the same lineage 3 as the RotaTeq-WI79-9 strain (Fig 3). The G1 strains B05-0442 and B05-0626 (lineage 2) shared high sequence similarity with Asian strains. All G2 strains were found in lineage 4, whereas RotaTeq-SC2-9 is in lineage 2. They had the closest relation to other G2 strains from Benin, Nigeria and Cameroon. The G3 strain B05-0647 (lineage 3) also was distinct from RotaTeq-WI78-8 (lineage 2). The two G8P[14] strains B05-0746 and B06-059 shared less than 94% identity with the closest strains from Sudan (MRC-DPRU447) and Kenya (LWK126). B06-0856 clustered with other G8P[4] strains from Kenya and Rwanda. All G12 strains were of lineage 3 and had the closest relation to strains from Benin, Brazil and Gabon. B06-0899 shared less than 95% identity with the closest other human G29 strains found in GenBank and 96% with the strain from buffalo (4426) (Fig 3). Except for BEF06018 (Belgium), all G29 strains were from African countries. The G29 strains were associated with different P types (P[6], P[14] and P[41]).

### NoV genetic diversity

From the 36 NoV positive samples, 32 belonged to genogroup GII, 3 to genogroup GI and one to both genogroups. We obtained RdRp sequences from 27 samples and capsid sequences from 20 samples (19 samples had both sequences). Based on both RdRp and capsid sequences, eight different genotypes were identified among NoV GII including the rare genotype GII.PNA7-GII.16 (Table 4 and Fig 2A and 2B). Overall, the common recombinant GII.P31-GII.4 Sydney strain was predominant with a detection rate of 28.57%. Out of the 3 NoV GI samples, one sample could be successfully genotyped as GI.1 based on the capsid sequence. In addition, we obtained the RdRp sequence from the sample co-infected with both genogroups and could assign the GII genotype to GII.P31 (Table 4).

**Table 4:** NoV polymerase and capsid genotypes.

Genogroup	RdRp based genotype	Capsid based genotype	Number (%)
**GI**		GI.1	1 (3.57%)
**GII**	GII.P16	GII.2	2 (7.14%)
	GII.P31	GII.4	8 (28.57%)
	GII.P16	GII.5	2 (7.14%)
	GII.P7	GII.6	2 (7.14%)
	GII.P8	GII.8	1 (3.57%)
	GII.P16	GII.13	2 (7.14%)
	GII.P7	GII.14	1 (3.57%)
	GII.PNA7	GII.16	1 (3.57%)
	GII.P16		1 (3.57%)
	GII.P7		3 (10.71%)
	GII.P30		2 (7.14%)
	GII.P31^£^		2 (7.14%)

£ one of the two GII.P31 genotypes detected originates from a mixed GI & GII infection

**Fig 4:**
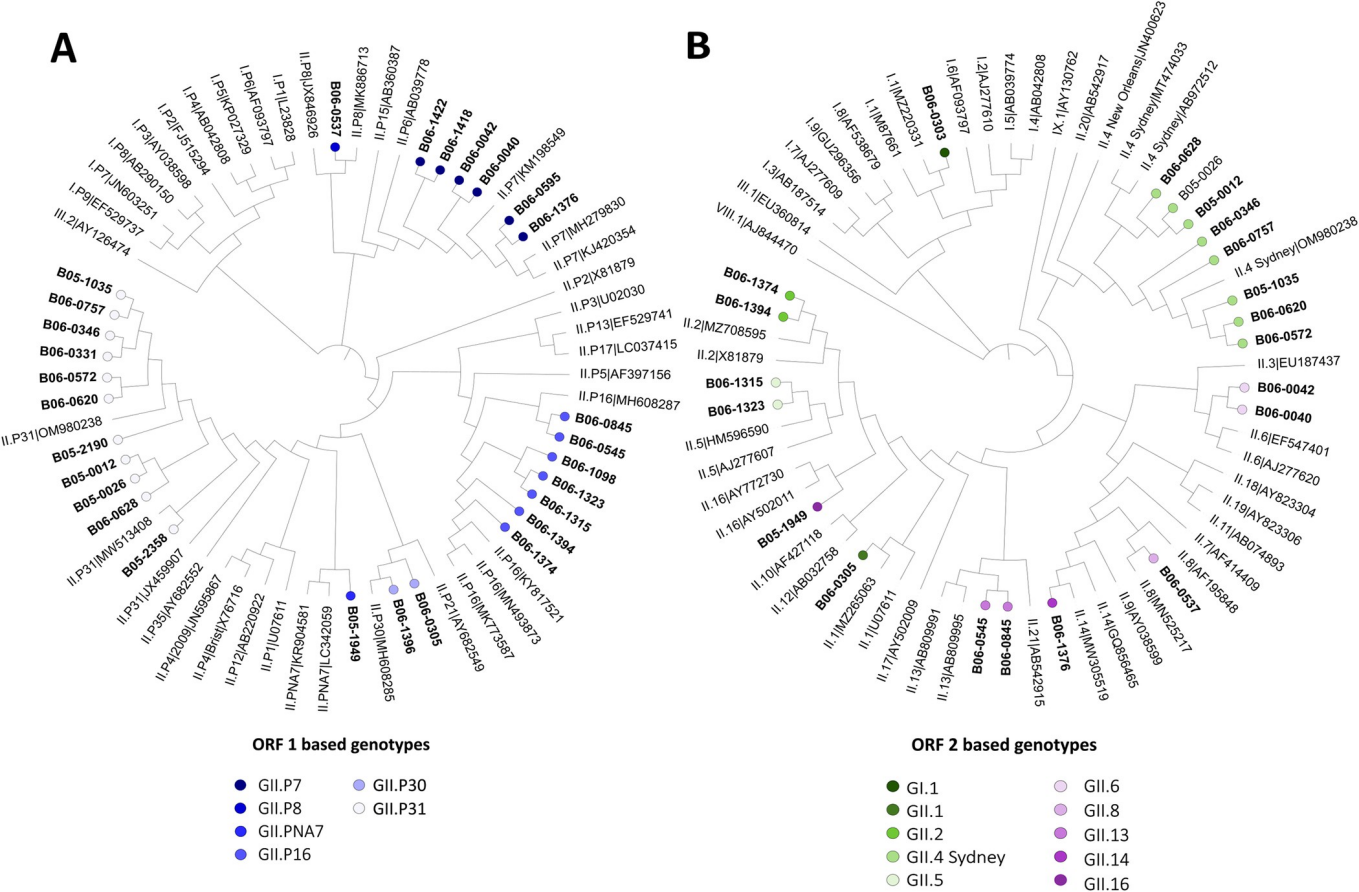
Phylogenetic analyses of ORF1 and ORF2 sequences from NoV strains from Burkina Faso. A. Phylogenetic analysis of 264 nt sequences of the RdRp gene (ORF1) from NoV positive samples (in bold) and human NoV reference sequences. B. Phylogenetic analysis of 585 nt sequences of the VP1 capsid gene (ORF2) from NoV positive samples (in bold) and human NoV reference sequences. Distinct color circles indicate the genotypes of strains used in this study.

The ORF1 sequences’ identity ranged between 65.02 and 100%, with the highest nucleotide distance being observed between the ORF1 genotypes GII.P7 and GII.P31 (Fig 4A). The sequences of the most frequently detected ORF1 genotype GII.P31 showed 94.52 to 100% nucleotide identity. The samples B06-0545 and B06-0845, both GII.P16-GII.13, cluster separately from the ORF1 sequences of the genotypes GII.P16-GII.5 (B06-1315, B06-1098) and GII.P16-GII.2 (B06-1374, B06-1394) (Fig 4A). In the ORF2-based genotype assignation, genotype GII.P16 ORF1-based sequences segregated in three different clusters (Fig 4B).

### HAstV genetic diversity

**Fig 5:**
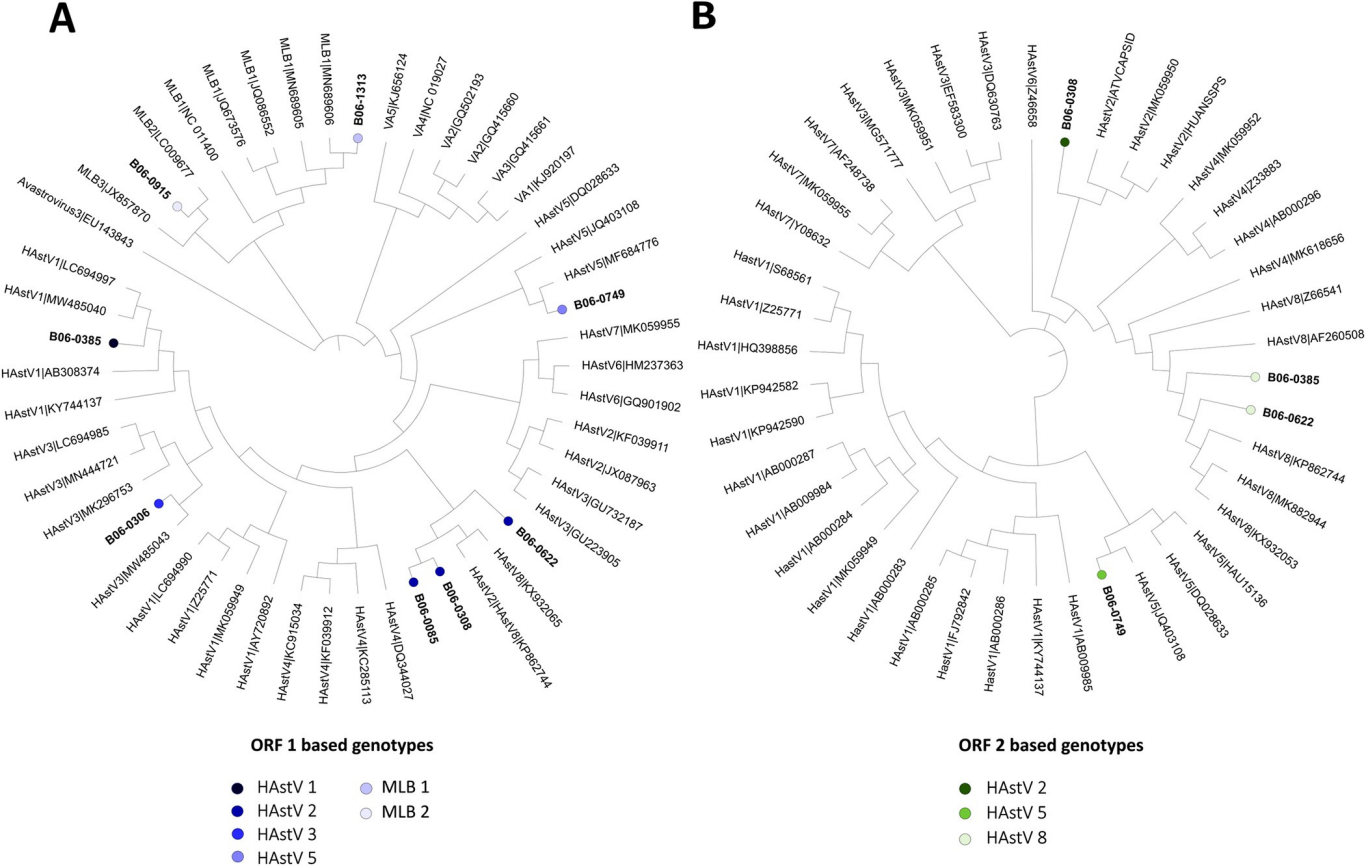
Phylogenetic analyses of ORF1 and ORF2 sequences from HAstV strains from Burkina Faso. A. Phylogenetic tree based on ORF1b sequences (363–488 nt) from HAstV positive samples (in bold) and HAstV reference sequences. B. Phylogenetic tree based on ORF2 sequences (331–334 nt) from HAstV classic strains (in bold) and HAstV reference sequences. Distinct color circles indicate the genotypes of strains used in this study.

We obtained partial sequences of ORF1b region for 7 HAstV positive samples (Fig 5A). The detected strains belonged to 5 different genotypes. Classic genotypes were predominant with 3 samples assigned to HAstV-2, one to HAstV-5, and one to HAstV-3 genotype. We suspected recombination events in two samples, one was assigned to HAstV-1 based on the ORF1 region and HAstV-8 based on the ORF2 region; the other was assigned to HAstV-2 according to the ORF1 region and HAstV-8 according to the ORF2 region. We also detected one HAstV-MLB1 and one HAstV-MLB2 strain (Fig 5).

### SaV genetic diversity

**Fig 6:**
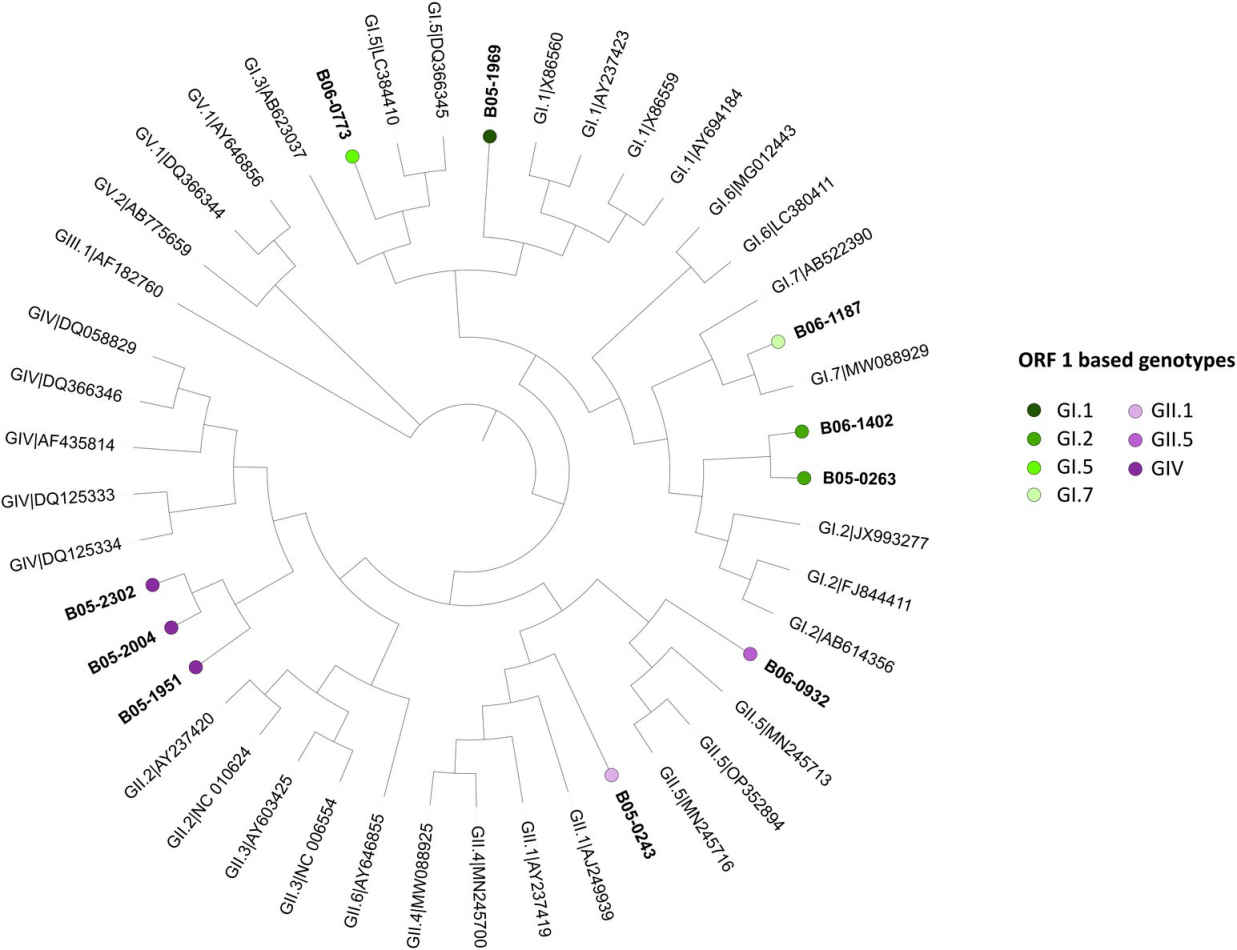
Phylogenetic analyses of ORF1 sequences from SaV strains from Burkina Faso. Phylogenetic tree based on RdRp sequences (584 nt, ORF1) from SaV positive samples (in bold) and human SaV reference sequences. Distinct color circles indicate the genotypes of strains used in this study.

Ten SaV positive samples were genotyped and assigned to 3 different genogroups based on their RdRp sequences (Fig 6). The most frequently detected genogroup was GI (n = 5), followed by GIV (n = 3) and GII (n = 2). In total six different genotypes could be identified: GI.2 in two samples and GI.1, GI.5, GI.7, GII.1 and GII.5 were detected each in one sample. The nucleotide sequence identity among all SaV sequences was between 62.28 and 99.69% with the highest sequence identity observed between sequences belonging to the GIV genogroup. Both GI.2 sequences showed a nucleotide identity of 91.11%.

## Discussion

The present study describes the detection and characterization of viruses associated with acute diarrhea in Burkinabe patients of all ages from urban and rural areas. One or more viruses were detected in 34.1% of the tested samples and children under 5 years were the most susceptible age group. Our detection rate is lower than previously observed by Ouedraogo et al., who found viral pathogens in 85.6% of symptomatic pediatric patients from the capital; however, they screened for AdV and Aichivirus in addition to the viruses presented here and prior to the RVA vaccination introduction [5]. The lower detection rates we observe can reflect the improvements in hygiene and access to drinking water made in the last years but can also be explained by differences in the study populations (age, urban or rural area), the detection methods used (immunoenzymatic and/or molecular biology) and the sampling period. It should also be noted that most studies to date focused on pediatric patients in limited periods of time, whereas we investigated symptomatic patients of all ages during four consecutive years. Importantly, detection rates may have been impaired by the un-even sampling during the study period. For instance, political unrests and the COVID-19 pandemic had a major impact on enrollments (e.g. no stool samples were collected during the COVID-19 confinement measures in April and May 2020, hospital and laboratory supply chains were disrupted). As previously described, we observed higher odds ratios for viral gastroenteritis for the under 5 years of age group [19]. However, patients declaring self-reported fever in the past 10 days before enrolment were associated with lower odds ratios, which could be due a later presentation to the health facilities, possibly after viral clearance. Hospitalization was also associated with lower odds ratios, suggesting milder manifestations of viral gastroenteritis compared to other causes. For instance, high fever, abdominal pain and bloody diarrhea are be more common in bacterial gastroenteritis [20,21].

We detected RVA among 8.4% of our cases, differently from studies before and after the introduction of the RotaTeq vaccine with prevalence ranging from 32.4 to 70% [5,6] and 14% respectively [6]. We observed the seasonal patterns described for RVA in BFA with peaks in infections during the dry and colder season (December to February) [22]. Moreover and in accordance with previous study in BFA, we confirm that RVA is no longer the leading cause of viral gastro-intestinal infection in BFA [6].

We observed a high genetic diversity of RVA with six G-genotypes and four P-genotype in eight different G/P combinations. The predominant G/P genotypes found here were G2P[4] and G12P[6] in agreement with a previous study conducted in BFA after the introduction of the RVA vaccine [23]. We therefore confirm the shift from G12P[8] and G6P[6] which were predominant prior to 2013. The low frequency of Wa-like P[8] strains and high detection rate of DS-1-like strains (genotypes P[4] and P[6]) are in line with several studies showing a shift towards DS-1-like strains [24,25]. Modelling of possible changes in the circulation of RVA genotypes after vaccination showed that the frequency heterotypic RVA strains would most likely increase [26]. Except for the two G1P[8] strains, all RVA strains in the present study have different VP4 genotypes compared to the RotaTeq strains. More than 50% of the strains were also heterotypic with respect to the VP7 gene. This corroborates data from a previous study on RVA strains in 2015, shortly after introduction of mass vaccination in Burkina Faso [6], where G2 and G12 strains were most frequent and G1P[8] accounted for 15% of detected strains.

Live vaccination mimics natural infection, which does not induce full protection against reinfection with any RVA strain either, but the severity is reduced with every reinfection [27] or vaccine dose, respectively. Therefore, it is to be expected that homotypic strains like are still being detected and not fully replaced, even after vaccine introduction. However, no strain from this study shared the same lineage as the RotaTeq strains. We also detected in a 16-month-old patient from a rural area, an uncommon RVA G29P[14] strain first described in African buffalos [28]. A GenBank search revealed only three previously described G29 strains in humans and species from the *bovinae* subfamily. P[14] strains are frequently detected as interspecies transmission events in humans [28,29]. Therefore, the G29P[14] detected may have been acquired through direct zoonotic transmission or transmission of a reassortant strain. Recent reassortment with circulating human RVA strains was also implied by the high identity with other P[14] strains from this study.

We observed an overall NoV detection rate of 14.1%, lower than the average 20% described in BFA [5,6]. GII was detected in 10.5% and GI in 3.6% of patients. Similarly to previous studies, NoV GII was found predominant [6] with GII.4 being the most common genotype [3,6]. We also detected a rare NoV GII.PNA7-GII.16 highlighting the diversity of NoV circulating strains. The ORF1 sequence from this strain has not yet been assigned to a genotype, as this virus has only been detected once in a patient from Japan [30].

We found HAstV in 6.8% of our patients, in line with reports from BFA and Côte d’Ivoire showing rates of 4.9% and 4% respectively [5,31]. HAstV was also the predominant virus found in samples co-infected with multiple enteric viruses. We detected the previously documented classic HAstV strains 2 and 5 [5,17]. However, we also detected the novel astroviruses strains MLB1 and MLB2 and two putative recombinant strains of classic genotypes (HAstV-1 and 8; 2 and 8) that have not been described before in BFA, which need further analysis. Studies conducted in Kenya, Gambia and Gabon have reported the circulation of the HAstV-VA genotypes [32] and HAstV-VA2 and MLB1 [17].

SaV was the second most detected virus, found in 9% of our cases in agreement with published detection rates of 18% and 10.3% [5,33]. We identified three SaV genogroups two of which, GI and GII, were previously described in BFA with a predominance of GI [5]. To our knowledge, we are the first to report the presence of genogroup GIV in BFA.

We show a decrease in the detection rates of viral agents responsible of gastroenteritis associated to a high genetic diversity with rare genotypes and recombinant viruses being reported for the first time in BFA. Our findings also highlight the potential for zoonotic transmission events and emergence of novel strains. Despite the improvements observed, diarrheal disease remains a major public health concern particularly among children. Moreover, the internal displacement of populations due to the current security situation in BFA may lead to reduced access to sanitation, hygiene, drinking water services, and potentially vaccines for refugees [34]. It should be noted that RVA is the only enteric virus routinely diagnosed in clinical settings in BFA. Therefore, the up-to-date epidemiological picture of enteric viruses presented here underlines the importance of routine testing and appropriate decisional algorithms for the management of viral gastroenteritis.

## Supporting information

S1 Fig ANDEMIA sentinel sites in Burkina Faso. The urban site of Bobo Dioulasso in the “Hauts-Bassins” region is indicated with a blue icon, the rural sites of Dano and Dissin in the “Sud-Ouest” region are indicated with light and dark green icons respectively. This map was generated in R using Leaflet (version 2.1.2) and Maps (version 3.4.2) packages with the U.S. Geological Survey (USGS) Imagery tile available from www.usgs.gov. The text and icons were added using Inkscape software.
(TIF)

S1 Table ANDEMIA patients sampling and screening results per month and year. (XLSX)

S2 Table GenBank accession numbers of the viral pathogens’ nucleotide sequences. (DOCX)

S3 Table Coinfections with enteric viruses. (DOCX)

S4 Table Enteric viruses’ detection rates by age group. (DOCX)

S5 Table Enteric viruses’ detection rates in urban and rural areas. (DOCX)

## References

[1] Rakau K.G., Nyaga M.M., Gededzha M.P., Mwenda J.M., Mphahlele M.J., Seheri L.M., Steele A.D., Genetic characterization of G12P[6] and G12P[8] rotavirus strains collected in six African countries between 2010 and 2014, BMC Infect. Dis. 21 (2021). 10.1186/s12879-020-05745-6. 33482744 PMC7821174

[2] Yuliya Zboromyrska J. Vila, Advanced PCR-based molecular diagnosis of gastrointestinal infections: challenges and opportunities, Expert Rev. Mol. Diagn. 16 (2016) 631–640. 10.1586/14737159.2016.1167599. 26986537

[3] Elbashir I.E., Aldoos N.F., Mathew S.M., Al Thani A.A., Emara M.M., Yassine H.M., Molecular epidemiology, genetic diversity, and vaccine availability of viral acute gastroenteritis in the middle East and North Africa (MENA) region, J. Infect. Public Health. 15 (2022) 1193–1211. doi: 10.1016/j.jiph.2022.09.001 36240530

[4] Burkina Faso climate: average weather, temperature, rain—Climates to Travel, (n.d.). https://www.climatestotravel.com/climate/burkina-faso (accessed July 3, 2023).

[5] Ouédraogo N., Kaplon J., Bonkoungou I.J.O., Traoré A.S., Pothier P., Barro N., et al. Prevalence and genetic diversity of enteric viruses in children with diarrhea in Ouagadougou, Burkina Faso, PLoS One. 11 (2016) 1–22. doi: 10.1371/journal.pone.0153652 27092779 PMC4836733

[6] Rönnelid Y., Bonkoungou I.J.O., Ouedraogo N., Barro N., Svensson L., Nordgren J., Norovirus and rotavirus in children hospitalised with diarrhoea after rotavirus vaccine introduction in Burkina Faso, Epidemiol. Infect. 148 (2020) e245. doi: 10.1017/S0950268820002320 32998792 PMC7592103

[7] Rotavirus Classification Working Group: RCWG—Laboratory of Viral Metagenomics, (n.d.). https://rega.kuleuven.be/cev/viralmetagenomics/virus-classification/rcwg (accessed October 31, 2022).

[8] Troeger C., Khalil I.A., Rao P.C., Cao S., Blacker B.F., Ahmed T., et al. Rotavirus Vaccination and the Global Burden of Rotavirus Diarrhea Among Children Younger Than 5 Years, JAMA Pediatr. 172 (2018) 958–965. doi: 10.1001/jamapediatrics.2018.1960 30105384 PMC6233802

[9] Burkina Faso: WHO and UNICEF estimates of immunization coverage: 2021 revision, (n.d.). https://cdn.who.int/media/docs/default-source/country-profiles/immunization/2022-country-profiles/immunization_bfa_2022.pdf?sfvrsn=6ec6afd2_3&download=true (accessed June 5, 2023).

[10] Bartsch S.M., Lopman B.A., Ozawa S., Hall A.J., Lee B.Y., Global economic burden of norovirus gastroenteritis, PLoS One. 11 (2016) 1–16. doi: 10.1371/journal.pone.0151219 27115736 PMC4846012

[11] Villabruna N., Koopmans M.P.G., de Graaf M., Animals as reservoir for human norovirus, Viruses. 11 (2019) 478. doi: 10.3390/v11050478 31130647 PMC6563253

[12] Oka T., Wang Q., Katayama K., Saifb L.J., Comprehensive review of human sapoviruses, 28 (2015) 32–53. doi: 10.1128/CMR.00011-14 25567221 PMC4284302

[13] Magwalivha M., Kabue J.P., Traore A.N., Potgieter N., Prevalence of Human Sapovirus in Low and Middle Income Countries, Adv. Virol. 2018 (2018). doi: 10.1155/2018/5986549 30245718 PMC6139206

[14] Astroviridae | ICTV, (n.d.). https://ictv.global/report_9th/RNApos/Astroviridae (accessed March 22, 2023).

[15] Schubert G., Achi V., Ahuka S., Belarbi E., Bourhaima O., Eckmanns T., et al. The African Network for Improved Diagnostics, Epidemiology and Management of common infectious Agents, BMC Infect. Dis. 21 (2021) 1–10. doi: 10.1186/S12879-021-06238-W/TABLES/2 34098893 PMC8184052

[16] Marques A.M., Diedrich S., Huth C., Schreier E., Group A rotavirus genotypes in Germany during 2005/2006, Arch. Virol. 152 (2007) 1743–1749. doi: 10.1007/s00705-007-0998-x 17557132

[17] Manouana G.P., Nguema-Moure P.A., Ngwese M.M., Bock C.T., Kremsner P.G., Borrmann S., et al. Genetic Diversity of Enteric Viruses in Children under Five Years Old in Gabon Gédéon, Viruses. 13 (2021). doi: 10.3390/v13040545 33805214 PMC8064335

[18] Niendorf S., Mas Marques A., Bock C.T., Jacobsen S., Diversity of human astroviruses in Germany 2018 and 2019, Virol. J. 19 (2022). doi: 10.1186/s12985-022-01955-3 36544187 PMC9773458

[19] Kotloff K.L., Nataro J.P., Blackwelder W.C., Nasrin D., Farag T.H., Panchalingam S., et al. Burden and aetiology of diarrhoeal disease in infants and young children in developing countries (the Global Enteric Multicenter Study, GEMS): A prospective, case-control study, Lancet. 382 (2013) 209–222. doi: 10.1016/S0140-6736(13)60844-2 23680352

[20] Luo L., Gu Y., Wang X., Zhang Y., Zhan L., Liu J., et al. Epidemiological and clinical differences between sexes and pathogens in a three-year surveillance of acute infectious gastroenteritis in Shanghai, Sci. Reports 2019 91. 9 (2019) 1–9. doi: 10.1038/s41598-019-46480-6 31292502 PMC6620335

[21] Holtz L.R., Neill M.A., Tarr P.I., Acute Bloody Diarrhea: A Medical Emergency for Patients of All Ages, Gastroenterology. 136 (2009) 1887–1898. doi: 10.1053/j.gastro.2009.02.059 19457417

[22] Ouedraogo N., Ngangas S., Bonkoungou I., Tiendrebeogo A., Traore K., Traore A., et al. Temporal distribution of gastroenteritis viruses in Ouagadougou, Burkina Faso: seasonality of rotavirus, BMC Public Health. 17 (2017). doi: 10.1186/s12889-017-4161-7 28327111 PMC5359802

[23] Bonkoungou I.J.O., Ouédraogo N., Tamini L., Teguera R.K., Yaméogo P., Drabo M.K., et al. Rotavirus and norovirus in children with severe diarrhea in Burkina Faso before rotavirus vaccine introduction, J. Med. Virol. 90 (2018) 1453–1460. doi: 10.1002/jmv.25213 29718582

[24] Mhango C., Mandolo J.J., Chinyama E., Wachepa R., Kanjerwa O., Malamba-Banda C., et al. Rotavirus Genotypes in Hospitalized Children With Acute Gastroenteritis Before and After Rotavirus Vaccine Introduction in Blantyre, Malawi, 1997–2019, J. Infect. Dis. 225 (2022) 2127–2136. doi: 10.1093/infdis/jiaa616 33033832 PMC9200156

[25] Donato C.M., Roczo-Farkas S., Kirkwood C.D., Barnes G.L., Bines J.E., Rotavirus Disease and Genotype Diversity in Older Children and Adults in Australia, J. Infect. Dis. 225 (2022) 2116–2126. doi: 10.1093/infdis/jiaa430 32692812 PMC9200153

[26] Pitzer V.E., Patel M.M., Lopman B.A., Viboud C., Parashar U.D., Grenfell B.T., Modeling rotavirus strain dynamics in developed countries to understand the potential impact of vaccination on genotype distributions, Proc. Natl. Acad. Sci. U. S. A. 108 (2011) 19353–19358. doi: 10.1073/pnas.1110507108 22084114 PMC3228484

[27] Velázquez F.R., Matson D.O., Calva J.J., Guerrero M.L., Morrow A.L., Carter-Campbell S., et al. Rotavirus infection in infants as protection against subsequent infections, N. Engl. J. Med. 335 (1996) 1022–1028. doi: 10.1056/NEJM199610033351404 8793926

[28] Strydom A., Donato C., Peenze I., Potgieter A.C., Seheri M., O’Neill H.G., Genetic characterisation of novel G29P[14] and G10P[11] rotavirus strains from African buffalo, Infect. Genet. Evol. 85 (2020). 10.1016/J.MEEGID.2020.104463. 32693063

[29] Strydom A., João E.D., Motanyane L., Nyaga M.M., Christiaan Potgieter A., Cuamba A., et al. Whole genome analyses of DS-1-like Rotavirus A strains detected in children with acute diarrhoea in southern Mozambique suggest several reassortment events, Infect. Genet. Evol. 69 (2019) 68–75. doi: 10.1016/j.meegid.2019.01.011 30641151

[30] Amarasiri M., Utagawa E., Sano D., Katayama K., Identification of novel norovirus polymerase genotypes from pediatric fecal samples collected between the year 1997 and 2000 in Japan, 82 (2020). https://pubmed.ncbi.nlm.nih.gov/32259662/ (accessed November 9, 2022). doi: 10.1016/j.meegid.2020.104313 32259662

[31] Bini J.C., Ekaza E., Faye-Kette H., Veh K.A., Nigue L., Borget-Alloue M.Y., et al. [Detection by RT-PCR of the 1st cases of Astrovirus in human stools in Abidjan, Côte d’Ivoire]., 100 (2007) 243–5. https://www.mendeley.com/catalogue/7dc68188-a1c9-3218-845d-83eaeec773f6/?utm_source=desktop&utm_medium=1.19.8&utm_campaign=open_catalog&userDocumentId=%7B44a8478c-8a83-3daf-aa17-9dabf29cc3bc%7D (accessed February 22, 2023). 17982851

[32] Meyer C.T., Bauer I.K., Antonio M., Adeyemi M., Saha D., Oundo J.O., et al. Prevalence of classic, MLB-clade and VA-clade Astroviruses in Kenya and the Gambia Emerging viruses, Virol. J. 12 (2015). doi: 10.1186/s12985-015-0299-z 25975198 PMC4465002

[33] Matussek A., Dienus O., Djeneba O., Simpore J., Nitiema L., Nordgren J., Molecular characterization and genetic susceptibility of sapovirus in children with diarrhea in Burkina Faso, Infect. Genet. Evol. 32 (2015) 396–400. doi: 10.1016/j.meegid.2015.03.039 25847694

[34] Burkina Faso | Rapports de situation, (n.d.). https://reports.unocha.org/fr/country/burkina-faso/ (accessed June 12, 2023).

