# Supplementary figures and images for: Genetic diversity of enteric viruses responsible of gastroenteritis in urban and rural Burkina Faso
Source: PLoS Negl Trop Dis. 2024 Jul 8;18(7):e0012228. doi: 10.1371/journal.pntd.0012228 (PMC11230633; doi:10.1371/journal.pntd.0012228)

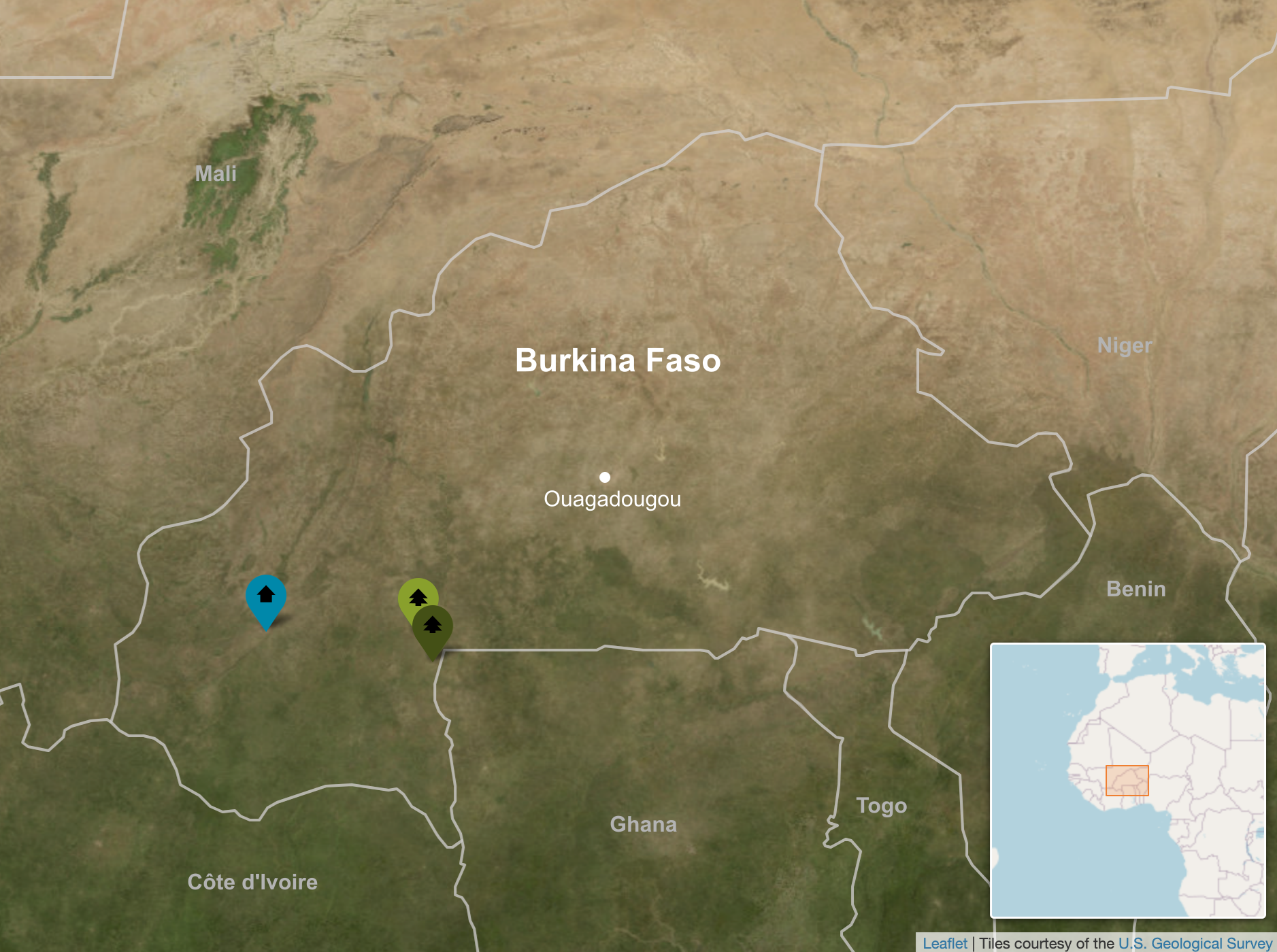

Supplement: S1 Fig — The urban site of Bobo Dioulasso in the “Hauts-Bassins” region is indicated with a blue icon, the rural sites of Dano and Dissin in the “Sud-Ouest” region are indicated with light and dark green icons respectively. This map was generated in R using Leaflet (version 2.1.2) and Maps (version 3.4.2) packages with the U.S. Geological Survey (USGS) Imagery tile available from www.usgs.gov. The text and icons were added using Inkscape software. (TIF) [file pntd.0012228.s001.tif]
