# Supplementary material for: Genetic diversity of enteric viruses responsible of gastroenteritis in urban and rural Burkina Faso
Source: PLoS Negl Trop Dis. 2024 Jul 8;18(7):e0012228. doi: 10.1371/journal.pntd.0012228 (PMC11230633; doi:10.1371/journal.pntd.0012228)
Supplement: S2 Table — (DOCX) [file pntd.0012228.s003.docx]

**S2 Table. GenBank accession numbers of the viral pathogens’ nucleotide sequences**

| **Taget** | **GenBank accession number** |
| --- | --- |
| HAstV (ORF1b) | OR427978-OR427984 |
| HAstV (ORF2) | OR427978-OR427984 |
| NoV (ORF1) | OR419692-OR419719 |
| NoV (ORF2-P2) | OR420025-OR420042, OR420074, OR420075 |
| RVA (VP4) | OR460953-OR460968 |
| RVA (VP7) | OR460969-OR460987 |
| SaV (ORF1) | OR452165-OR452174 |
