# Supplementary material for: Genetic diversity of enteric viruses responsible of gastroenteritis in urban and rural Burkina Faso
Source: PLoS Negl Trop Dis. 2024 Jul 8;18(7):e0012228. doi: 10.1371/journal.pntd.0012228 (PMC11230633; doi:10.1371/journal.pntd.0012228)
Supplement: S3 Table — (DOCX) [file pntd.0012228.s004.docx]

**S3 Table. Coinfections with enteric viruses**

| **Viral combinations** | **Number detected** |
| --- | --- |
| RVA + SaV | 7 |
| HAstV + SaV | 7 |
| HAstV + NoV GII | 7 |
| HAstV + RVA | 6 |
| NoV GII + SaV | 5 |
| NoV GI + SaV | 4 |
| NoV GI + NoV GII | 4 |
| HAstV + NoV GI | 4 |
| NoV GII + RVA | 2 |
| NoV GI + RVA | 1 |
| HAstV + NoV GI + NoV GII | 1 |
| NoV GI + NoV GII + SaV | 1 |
| HAstV + RVA + SaV | 1 |
| Total | 50 |
