# Supplementary material for: Genetic diversity of enteric viruses responsible of gastroenteritis in urban and rural Burkina Faso
Source: PLoS Negl Trop Dis. 2024 Jul 8;18(7):e0012228. doi: 10.1371/journal.pntd.0012228 (PMC11230633; doi:10.1371/journal.pntd.0012228)
Supplement: S4 Table — (DOCX) [file pntd.0012228.s005.docx]

**S4 Table. Enteric viruses’ detection rates by age group**

|  | **Age groups** | | |  |
| --- | --- | --- | --- | --- |
| **Virus** | **< 5 yo N (%)** | **5-15 yo N (%)** | **>15 yo N (%)** | **p-value** |
|  | (N=1148) | (N=47) | (N=100) |  |
| **HAstV** | 87 (7.6%) | 1 (2.1%) | 0 (0%) | 0,001* |
| **RVA** | 107 (9.3%) | 1 (2.1%) | 1 (1%) | 0.001* |
| **NoV GI** | 45 (3.9%) | 0 (0%) | 2 (2%) | 0.441 |
| **NoV GII** | 127 (11.1%) | 2 (4.3%) | 7 (7%) | 0.202 |
| **SaV** | 109 (9.5%) | 1 (2.1%) | 4 (4%) | 0.047* |

***** Indicates a statistically significant p-value (Fischer’s exact test)
