# Supplementary material for: Genetic diversity of enteric viruses responsible of gastroenteritis in urban and rural Burkina Faso
Source: PLoS Negl Trop Dis. 2024 Jul 8;18(7):e0012228. doi: 10.1371/journal.pntd.0012228 (PMC11230633; doi:10.1371/journal.pntd.0012228)
Supplement: S5 Table — (DOCX) [file pntd.0012228.s006.docx]

**S5 Table. Enteric viruses’ detection rates in urban and rural areas**

|  | **Residency** | |  |
| --- | --- | --- | --- |
| **Virus** | **Urban N (%)** | **Rural N (%)** | **p-value** |
|  | (N=315) | (N=980) |  |
| **HAstV** | 8 (2.5%) | 80 (8.2%) | 0.001* |
| **RVA** | 48 (15.2%) | 61 (6.2%) | <0.001* |
| **NoV GI** | 7 (2.2%) | 40 (4.1%) | 0.261 |
| **NoV GII** | 33 (10.5%) | 103 (10.5%) | 0.986 |
| **SaV** | 13 (4.1%) | 101 (10.3%) | 0.001* |

***** Indicates a statistically significant p-value (Pearson χ2 test)
